# 360° size-adjustable microelectrode array system for electrophysiological monitoring of cerebral organoids

**DOI:** 10.3389/fbioe.2025.1596009

**Published:** 2025-07-22

**Authors:** Takashi Ozaki, Norikazu Ohta, Jiaju Ma, Minoru Hirano

**Affiliations:** Frontier Management Office, Toyota Central R&D Labs. Inc., Nagakute, Japan

**Keywords:** bioelectric potentials, electrophysiology, microelectrodes, organoids, brain organoids

## Abstract

This paper presents a 360°, size-adjustable microelectrode array (MEA) system for the long-term electrophysiological monitoring of cerebral organoids derived from human pluripotent stem cells. The system consists of eight independently positionable multielectrode probes, each carrying eight electrodes arranged vertically. This configuration resulted in 64 recording channels surrounding the organoid. The multielectrode probes were mounted on custom-designed miniature manipulators with three degrees of freedom. This setup enabled positioning and contact with organoids of varying sizes (approximately 1–3.7 mm in diameter). The design allowed circumferential access and facilitated standard incubator-based cultivation without disrupting the recording setup. Fabricated using flexible printed circuit technology, this MEA system offers relatively low production costs. It is also amenable to widespread implementation in laboratory settings. Experimental results demonstrated the successful recording of neuronal activity, including spike detection and signal stability, over 2 weeks of continuous organoid culture. These results suggests that the three-dimensional system provides broad spatial coverage and supports long-term monitoring for basic biomedical research. It also holds potential for future applications such as biohybrid computing.

## 1 Introduction

Recent studies have focused on three-dimensional (3D) neuronal cell cultures and cerebral organoids derived from human pluripotent stem cells (hPSCs). These models are increasingly explored as alternatives to animal models for studying electrophysiology and neuropathology (Zhuang et al., 2018). Human embryonic stem cell–based organoids have been employed to model the folding and gyrification of the human brain associated with neurodevelopmental disorders (Karzbrun et al., 2018). Additionally, well-defined neurogenesis in organoid-on-chip systems has been utilized to replicate the early stages of human brain development (Wang et al., 2018).

3D cerebral organoids exhibit structural and functional characteristics that more closely resemble those of human brain tissue compared to conventional two-dimensional (2D) cultures (Qian et al., 2019; Adlakha, 2023; Gu et al., 2024). This makes them increasingly valuable for investigating higher-order neuronal mechanisms. Furthermore, they are being explored as potential building blocks for biohybrid computing, where live neuronal networks could serve as energy-efficient, low-power alternatives to traditional electronic architectures. Previous studies have shown that organoid-based neural circuits can represent disease pathology and display both spontaneous and evoked activities (Sun et al., 2021; Zhang et al., 2023), emphasizing the need for precise and scalable electrophysiological monitoring in 3D systems. Meanwhile, recent developments in artificial intelligence have raised concerns regarding energy consumption and CO_2_ emissions (Budennyy et al., 2022; Kramer, 2024). In contrast, biological neuronal networks operate with significantly lower energy under comparable computational load. Consequently, research efforts are increasingly focused on integrating human brain organoids (Cai et al., 2023) and other neural networks (Kagan et al., 2022) into computing systems. These innovative approaches require stable and reliable electrophysiological measurements in 3D tissue models, which are critical for advancing fundamental neuroscience and could guide the development of low-energy computational architectures.

To understand and control the complex dynamics of 3D neural networks, direct measurement of electrical signals is essential. Traditional 2D microelectrode arrays (MEAs) have been extensively used for recording electrical signal measurements in neuronal cultures and brain slices, providing high temporal resolution and valuable insights into brain network functionality. For instance, 2D MEA systems have enabled frequency spectrum analyses of neuronal activity in cortical or hippocampal slices (Ito et al., 2014). Additionally, advancements in complementary metal-oxide-semiconductor technology have facilitated the development of versatile, large-scale 2D MEA devices with cellular resolution (Tsai et al., 2017). Even more recently, ultra-high-density CMOS platforms have achieved channel counts exceeding 26,000 (Andrews et al., 2024) and 236,000 (Li et al., 2025) electrodes, underscoring a trend toward massive parallelism. However, despite this impressive scaling, these CMOS devices remain single-sided and therefore inherit the same geometric mismatch with 3D spheroids described above (Supplementary Table S1). While transitioning from 2D to 3D culture models yields more physiologically representative structures, it simultaneously amplifies the challenges associated with electrophysiological recording and stimulation. Spheroid organoids, characterized by variable diameters, irregular shapes, and heterogeneous cellular compositions, demand electrode systems capable of adapting to these complexities. Moreover, electrodes must interface with multiple regions of the organoid surface and interior to capture the intricate network dynamics. Conventional 2D MEAs often fall short of these requirements, as they can contact only one side of the 3D tissue, resulting in limited spatial coverage and weaker signal acquisition from distal regions. Thus, an innovative MEA design that provides full angular access (for instance, 360° coverage), accommodates different organoid sizes, and supports long-term culture, is indispensable for advancing both fundamental research and applications such as bio-hybrid computing.

Consequently, 3D MEAs have been developed to address the limitations of traditional designs. For example, rigid planar electrodes have been arranged in stepped laminated structures (Musick et al., 2009); however, these designs fail to adapt to the diverse sizes and shapes of organoids. In contrast, flexible mesh-structured MEAs offer improved contact by embedding electrodes into organoids during their growth (McDonald et al., 2023). Nevertheless, these devices remain fundamentally 2D and are unable to capture signals from all directions. Truly 3D MEA designs that wrap around organoids have been explored, utilizing self-folding sheets driven by residual stress. For example, a star-shaped electrode structure can self-curve to envelope an organoid (Cools et al., 2018). Similarly, tube-like architectures that roll into a cylindrical shape have been proposed to encapsulate organoids (Kalmykov et al., 2019), while stretchable elastic substrates can contract around them (Park et al., 2021). A related approach uses highly stretchable bottom-side MEAs that conform to the basal surface of organoids (Kim et al., 2025), but such systems still leave the apical and lateral aspects unprobed and require specialized microwell holders (Supplementary Table S1). Although these approaches enable 3D measurements, each design is limited by its predefined size, making it challenging to accommodate the size and shape variations of individual organoids. This limitation can potentially lead to variability in measurement yield.

To address these challenges, we present a 360° 3D MEA system for electrophysiological monitoring and stimulation of cerebral organoids. This system consists of eight multielectrode probes, each equipped with eight vertically arranged microelectrodes, providing a total of 64 channels. These probes surround the organoid and allow insertion from all directions, offering complete angular coverage—a substantial advantage over existing designs. Each probe features three degrees of freedom for independent positioning, enabling precise and adaptable measurements. Additionally, the compact design supports extended cultivation within standard incubator. Utilizing flexible printed circuit (FPC) board technology, this platform is well-suited for large-scale production and practical application in research settings.

## 2 Methods

### 2.1 Design of electrode system

**FIGURE 1:**
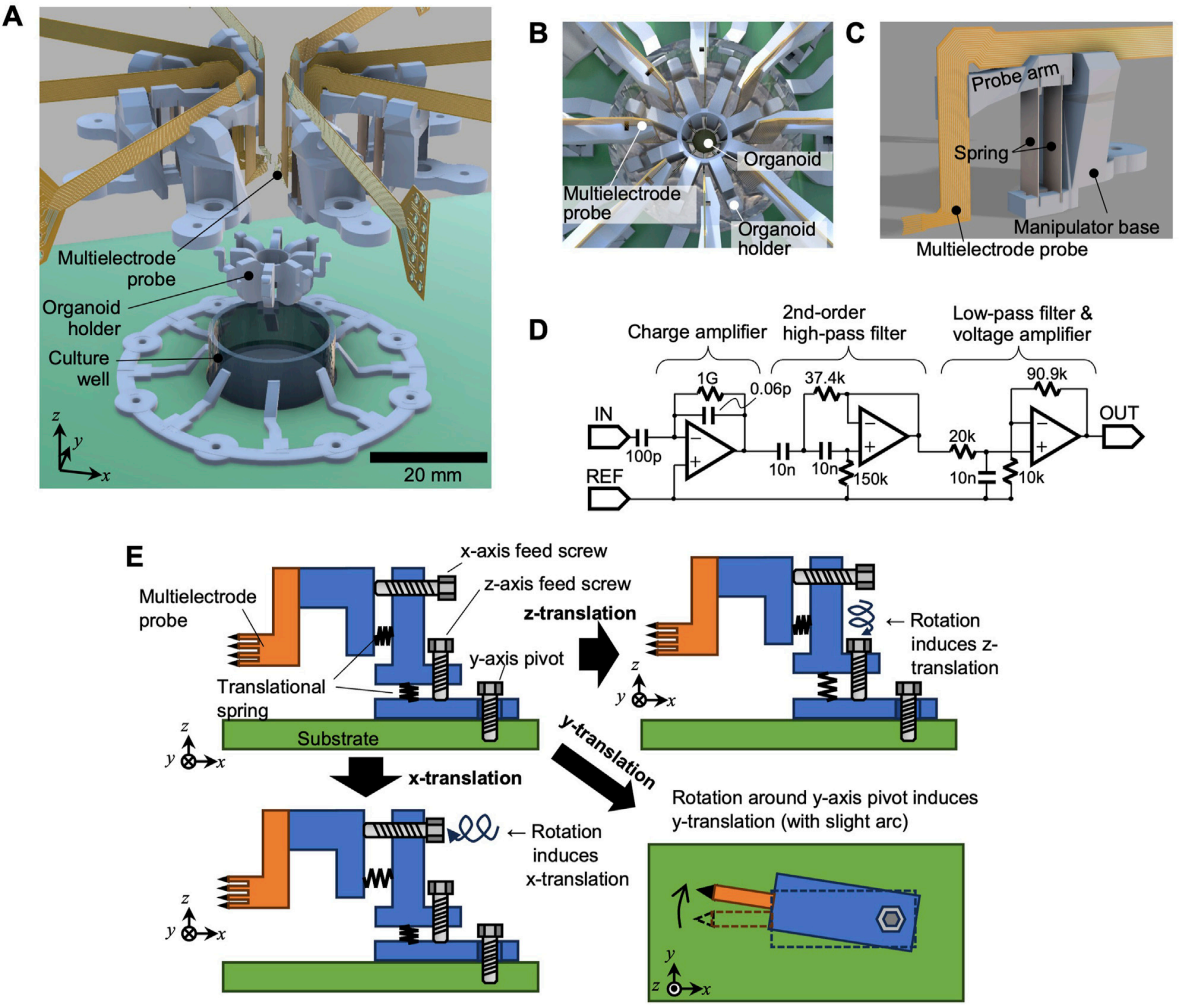
System overview. **(A)** Exploded view of the electrode system, **(B)** top view illustration with the organoid in place, **(C)** single multielectrode probe and its manipulator, and **(D)** circuit schematic for signal amplification. **(E)** Mechanism of the probe manipulator, illustrating independent adjustment along the x- and z-axes with rotation around the z-axis.

The primary mechanical components of the system include the culture well, organoid holder, and eight multielectrode probes (Figure 1A).

• Culture well: Filled with culture medium during operation to support organoid growth and viability.
• Organoid holder: The holder inserts into the culture well and provides a hollow cylindrical structure at its center (Figure 1B). The organoid was positioned within the cylinder, ensuring it remained centrally located in the well. Slits in the cylinder walls allowed the multielectrode probes to access the organoid from multiple directions.
• Multielectrode probe: Each probe featured a needle-like tip containing eight vertically aligned microelectrodes. Neuronal activity was recorded by pressing the probe tip against the organoids. A total of eight probes surrounded the organoids, resulting in 64 electrodes (8 × 8).

Each multielectrode probe was connected to a miniature positioning manipulator (Figure 1C), allowing for independent adjustment of its position (Detailed specifications are described below).

The signal lines from the probes were connected to a signal amplification circuit (Figure 1D) on a dedicated printed circuit board. The amplification included a charge amplifier in the first stage, followed by a second-stage Sallen–Key high-pass filter with a cut-off frequency of approximately 212 Hz. This was then followed by a voltage amplifier with a low-pass cutoff near 7.96 kHz and a gain of 10.1. An AD8606 amplifier (Analog Devices, Inc.) is employed in the circuit. To minimize noise, the circuit board was housed within an Al-shielded enclosure. In addition, the shielded enclosure was connected (grounded) to both the circuit ground and the reference electrode inside the culture well. The amplified signals were then acquired using a data acquisition module (USB-6363, National Instruments), which was controlled by a personal computer with a readout sampling rate of 25 kHz. Figure 1E illustrates the design of the probe manipulator. Translation was provided along the x- and z-axes using lead screws, enabling linear movement, as depicted in the lower-left and upper-right parts of Figure 1E, respectively. For adjustments along the y-axis direction, the entire manipulator rotates around the z-axis.

### 2.2 Culturing cerebral organoids

#### 2.2.1 Undifferentiated culture of human-induced pluripotent stem cells (hiPSCs)

Human mesenchymal stromal cell-derived hiPSC clones (Alstem, Inc., #iPS12) reprogrammed using an episomal vector, were purchased from Filgen, Inc. Undifferentiated cultures and differentiation media for cerebral organoids were obtained from StemCell Technologies. Briefly, hiPSCs were maintained in mTeSR™ Plus medium, which was replaced daily except on days 5 and 6. Cells were passaged using the colony dissociation method with ReLeSR™, typically at a dilution ratio of 1:50 to 1:150.

#### 2.2.2 Cerebral organoid formation and maturation

On the day of passage (day 7), cells were dissociated into single cells using Gentle Cell Dissociation Reagent. The cells were then prepared for cerebral organoid differentiation using the STEMdiff™ Cerebral Organoid Kit, following the instructions from the manufacturer. For embryoid body (EB) formation and initial neuroectoderm differentiation, dissociated cells were seeded into a 96-well round-bottom ultra-low attachment plate (Corning, #7007). Each well contained 9,000 cells in EB Formation Medium. The medium was replaced on days 2 and 4. On day 5, the EBs were transferred to a new 24-well flat ultra-low attachment plate (Corning, #3473). At this stage, the medium was changed to Induction Medium to promote neuroectoderm neuroepithelium formation. On day 7, EBs were embedded in Matrigel hESC-Qualified Matrix (Corning, # 354277). The embedded EBs were then transferred to 6-well Ultra-Low Attachment Plates (Corning, #3471) containing Expansion Medium. This step facilitated the expansion of neuroepithelial buds by EBs, providing a 3D extracellular matrix milieu. On day 10, the medium was replaced with Maturation Medium under shaking conditions at 65 rpm. The organoids were matured by replacing the medium every three to 4 days. Electrophysiological analysis was typically performed after 3–4 months of maturation. For the long-term recording experiments, three biologically independent organoids were used.

### 2.3 Statistics

All statistical analyses were performed unless otherwise specified. Data are presented as box plots showing the median, interquartile range (IQR), and whiskers extending to 1.5 times the IQR. A p-value of less than 0.05 was considered statistically significant. Raw electrophysiological data were post-processed by applying a digital band-pass filter (300–5000 Hz). Action potential spikes were detected using a threshold set at five times the standard deviation of the baseline noise. The effect of platinum black coating on electrode impedance was assessed using a paired t-test for each of the eight probes, with a Bonferroni correction applied to account for multiple comparisons (significance level set at p < 0.05/8). The influence of organoid size on signal-to-noise (S/N) ratio was evaluated using a two-sided Mann–Whitney U test. The effect size was quantified using Cliff’s delta (Δ). Functional connectivity was quantified using the spike-time tiling coefficient (STTC) with a synchronicity window (τ) of 10 ms. Channels with fewer than three detected spikes over the recording period were excluded from this analysis. The physical distance between electrodes was modeled as the three-dimensional Euclidean distance on the surface of an idealized cylinder. The relationship between inter-electrode distance and the probability of high synchrony (defined as STTC >0.5) was analyzed using a logistic regression model. For long-term recording stability, changes in S/N ratio and spike frequency over 2 weeks were assessed using the Wilcoxon signed-rank test on data pooled from three independent organoids. Effect sizes were calculated using Cliff’s delta (Δ), and the median difference was estimated using the Hodges–Lehmann estimator.

## 3 Results

### 3.1 Fabrication results

**FIGURE 2:**
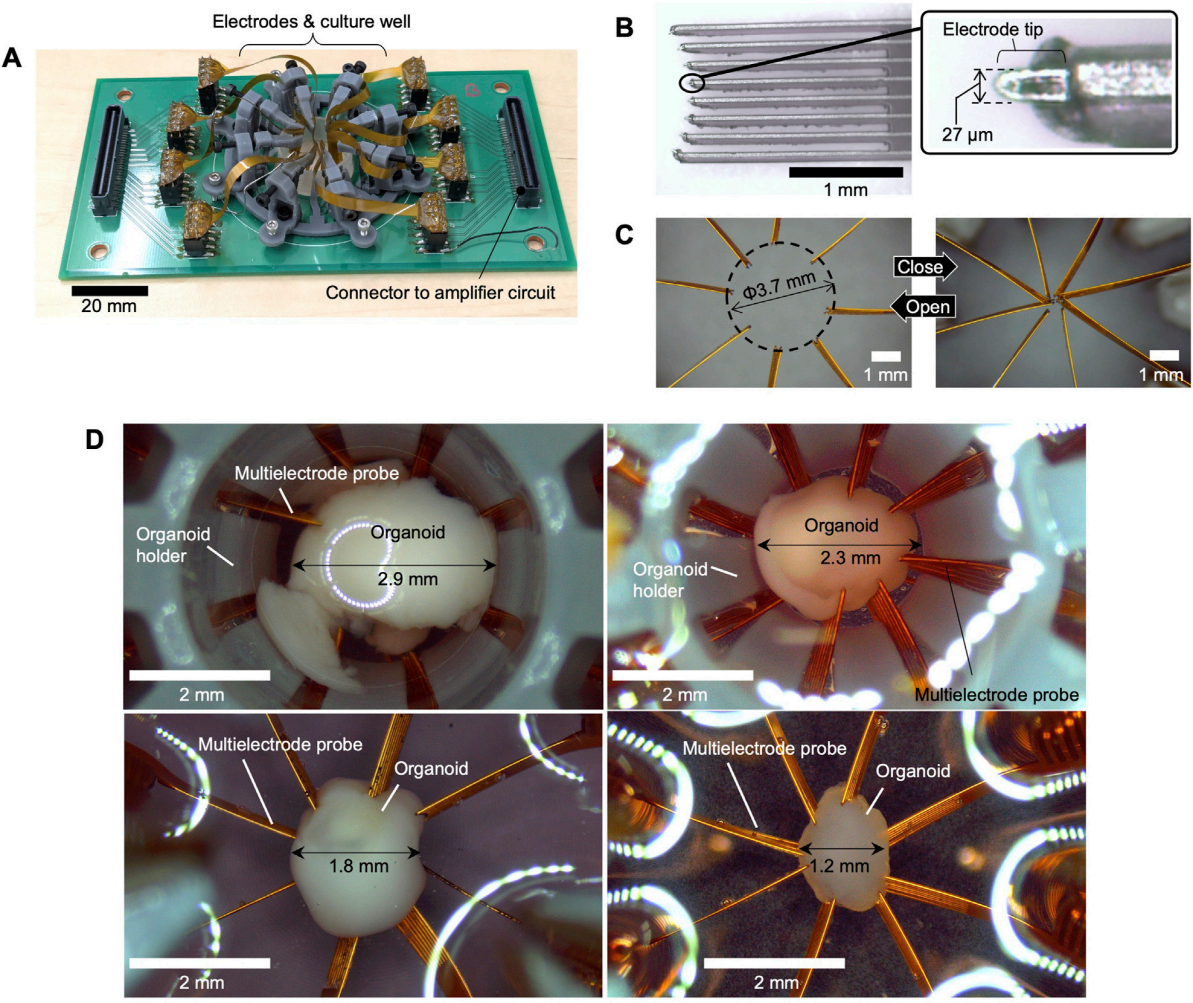
Fabrication results. **(A)** The entire system, **(B)** close-up of the multielectrode probe tip, **(C)** photographs showing the probes in open and closed positions. **(D)** Photographs of organoids inserted into the electrode system with different diameters.

Figure 2A illustrates the electrode system, which was built on a 140 mm × 70 mm board. A culture well was placed at the center, with connectors linking to the amplification circuit at both ends. The probe manipulators were fabricated using 3D printing (Form3, Formlabs) with Gray Resin V4. Figure 2B depicts the multielectrode probe, along with a magnified view of its tip. The probes were fabricated using FPC technology. To expose the electrode tip, Cu traces were extended from the FPC edge as “flying leads (unsupported Cu traces extended beyond the polyimide edge),” each measuring 27 µm in width. The eight microelectrodes were arranged in a slightly curved layout to match the spherical surfaces of the organoids. Figure 2C highlights the adjustable positioning of the multielectrode probes. The left image shows the probes in their maximum open position (positive x-axis direction), while the right image shows them in the fully closed position. Due to manufacturing variations, the probe tips do not align perfectly in the closed position; thus, organoids with diameters below 1 mm are difficult to measure. This prototype is suitable for organoids with diameters ranging from approximately 1–3.7 mm. Figure 2D presents the results of applying the system to organoids of various diameters (1.2–2.9 mm). In this procedure, the electrode probes were initially set to their fully open positions. The organoid was then placed at the center, and the probes were gradually moved inward until the electrodes made contact with the organoid. By adjusting the electrode opening through the positioning mechanism, the electrodes could be reliably inserted for organoids of different sizes, thereby demonstrating the feasibility of this system.

### 3.2 Electrical activity recording

**FIGURE 3:**
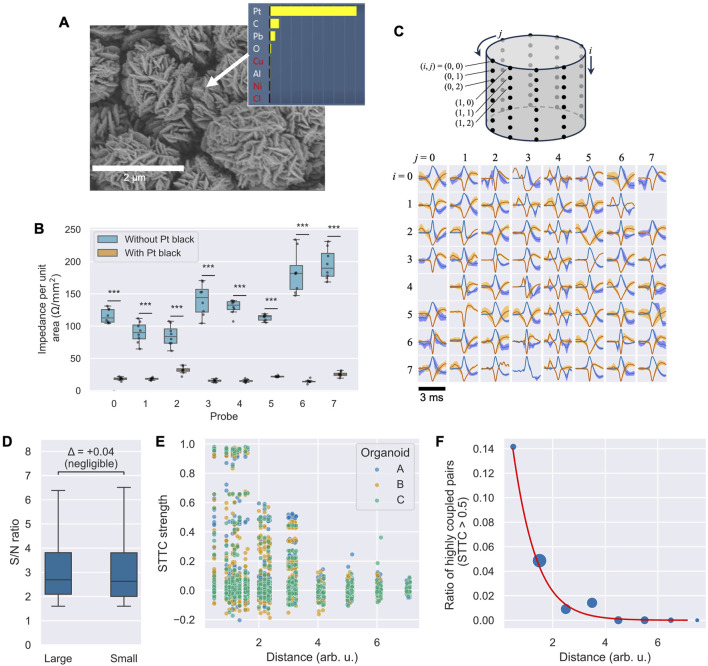
Electrode characterization and activity analysis. **(A)** SEM image and EDX elemental analysis confirming Pt black deposition. **(B)** Box plots of electrode impedance at 1 kHz for each of the eight probes before and after Pt black deposition (n = 8 electrodes per probe). All differences are statistically significant (paired t-test with Bonferroni correction for eight comparisons, $p<\frac{0.05}{8}$ ). **(C)** Representative spike waveforms recorded via the microelectrode array, with the top cylindrical illustration indicating vertical (*i*) and circumferential (*j*) electrode coordinates. **(D)** Box plots comparing the S/N ratio of detected spikes from organoids of different sizes (1.2 mm and 2.3 mm diameter; n = 64 channels per organoid). Cliff’s delta (Δ) indicates a negligible effect of size. **(E)** Scatter plot of spike train correlation (STTC) versus geometric distance between electrode pairs for three organoids **(A–C)**. **(F)** Probability of high synchrony (STTC >0.5) as a function of inter-electrode distance. The bubble size represents the number of pairs in each distance bin, and the red line shows the logistic regression fit.

Various electrode coatings have been proposed to reduce contact impedance with cells and enhance the signal strength of action potentials (Yang et al., 2020). One such coating is a Pt electrode with a nanostructure, known as platinum black (Pt black) (Zhang et al., 2015; Fan et al., 2020), which was employed in this study. Prior to use, Pt black was electrodeposited on the probe surfaces to reduce impedance between the electrodes and neurons. The deposition process utilized a Pt wire counter electrode, a solution of chloroplatinic acid (3 wt%) and lead acetate (0.1 wt%), a current density of 10 mA/cm^2^, and a process time of 7.5 min. A VI-207F1 power source (NF Corporation) was used for this procedure. Figure 3A presents a scanning electron microscopy (SEM) image and energy-dispersive X-ray (EDX) analysis of the Pt black surface, confirming its characteristic nanostructure and composition. Figure 3B illustrates the measured electrode impedance in culture medium, with and without Pt black, for each of the eight probes. The box plots show the distribution of impedance values from the eight electrodes on each probe. The average impedance decreased significantly for all eight probes after Pt black deposition (paired t-test, p < 0.05 for all comparisons), with some variability observed between probes. The overall average impedance across all electrodes decreased from 131.6 to 20.0 Ω/mm^2^, a reduction to approximately one-seventh of the original value.

After placing the organoids on the multielectrode probes, the signals were recorded for 5 minutes. Postprocessing involved applying a band-pass filter (300 Hz–5 kHz), followed by a threshold of five times the standard deviation to detect action potential spikes. Figure 3C showcases the average spike waveforms, normalized to the spike amplitude from each microelectrode. Positive spikes are depicted in blue, while negative spikes are shown in red. The shaded areas in light blue and orange indicate ±1 standard deviation of the detected spikes. Spikes exhibiting both positive and negative major deflections suggest signals originating from multiple neuronal sources. Of the 64 tested electrodes, 62 demonstrated clear spikes. The detection of signals by the majority of electrodes indicates stable contact between the organoid and the electrode probes upon insertion.

### 3.3 Influence of organoid size on signal quality

To assess whether organoid size affects the quality of the recorded signals, we compared the signal-to-noise (S/N) ratio of spikes from two organoids with different diameters (approximately 1.2 mm and 2.3 mm). A Mann–Whitney U test indicated a statistically significant difference between the two groups (U = 2.02 × 10^7^, p = 0.00021 < 0.05), likely due to the large sample size (n = 64 channels per group). However, the effect size was negligible (Cliff’s Δ = 0.04). As shown in the box plots in Figure 3D, the median S/N ratios were practically comparable. This suggests that our system can achieve consistent recording quality across a range of organoid sizes.

### 3.4 Network activity analysis

To demonstrate the system’s utility for network-level analysis, we investigated the relationship between functional connectivity and the physical distance between electrodes across three independent organoids. We calculated the spike-time tiling coefficient (STTC) for all possible electrode pairs to measure spike train correlation.

Figure 3E shows a scatter plot of the resulting STTC values against the geometric distance for all electrode pairs. A visual inspection of the plot shows a clear trend: while a majority of pairs exhibited low correlation (STTC ≈ 0), pairs with strong correlations appear to be concentrated at shorter physical distances, suggesting a relationship between network connectivity and spatial proximity. To quantify this observation, we analyzed the probability of observing a high-synchrony pair, defined as STTC > 0.5. As shown in Figure 3F, where bubble size represents the number of pairs in each distance bin, the likelihood of finding a highly synchronous pair decreased sharply with increasing distance. A logistic regression model confirmed a significant negative relationship between distance and the probability of high synchrony (slope coefficient = −1.26, p < 0.001). This result indicates that our system can resolve spatially dependent patterns of neural activity, making it a valuable tool for studying the functional architecture of 3D neural networks.

### 3.5 Feasibility of long-term recording

**FIGURE 4:**
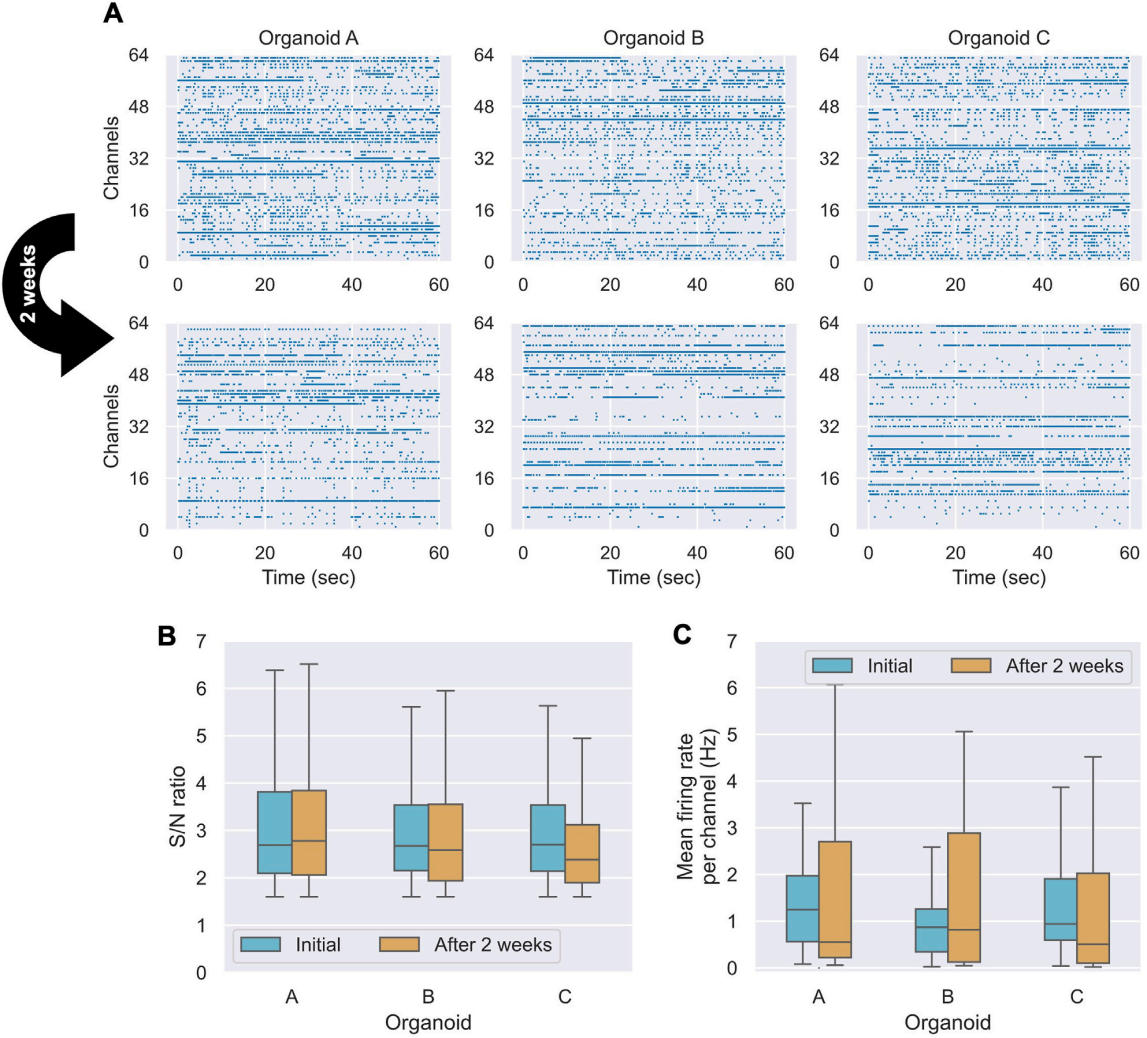
Comparison of measurements before and after 2 weeks of culture (n = 3 organoids). **(A)** Spike detection raster plots for three organoids **(A–C)** over a 60-s period at the initial time point and after 2 weeks. **(B)** Box plots of the S/N ratio distribution for each organoid. **(C)** Box plots comparing the spike frequency distributions for each organoid.

To evaluate the stability of our system for long-term measurements, three organoids (n = 3) were maintained in a CO_2_ incubator for 2 weeks while being continuously attached to the electrodes. We compared neuronal activity between the initial measurement and after 2 weeks. Figure 4A displays raster plots of spikes detected across all channels over a 60-s period for each of the three organoids at both time points. All organoids exhibited active spiking at both the initial and 2-week measurements, although the specific set of active channels sometimes shifted, reflecting the dynamic nature of the biological network. To quantify signal stability, we analyzed the S/N ratio and spike frequency across all recorded channels. The S/N ratio (Figure 4B) showed a statistically significant but negligible decrease over the 2-week period (Wilcoxon signed-rank test, p = 0.049; Cliff’s Δ = +0.039; Hodges–Lehmann median Δ = −0.029). This suggests that while there may be a subtle systematic change, the practical degradation in signal quality at the electrode-organoid interface was slight. In contrast, the overall spike frequency showed no statistically significant change (Wilcoxon signed-rank test, p = 0.475; Figure 4C), with a small effect size (Cliff’s Δ = +0.148), indicating that the overall firing activity of the networks was well-maintained. The individual changes observed in the raster plots (Figure 4A) likely represent the spontaneous reorganization of network activity, a hallmark of living neuronal cultures, rather than a systematic decline in viability. Taken together, these results demonstrate that our system provides a stable interface for long-term electrophysiological monitoring. The minimal degradation in S/N ratio and the sustained firing activity confirm its suitability for chronic studies of neuronal development and plasticity in 3D organoid models.4 Discussion.

Regarding the electrode arrangement, for an organoid with a 3-mm diameter, the eight multielectrode probes positioned around the circumference result in an interprobe spacing of approximately 1.18 mm along the circumference (2πR/8). In contrast, a 2D MEA covering an equivalent planar area typically operates with a smaller electrode spacing, such as 0.25 mm when 8 × 8 electrodes are arranged in a 2-mm square. The wider spacing in the proposed system may help reduce signal overlap among electrodes and improve the spatial independence of recording and stimulation sites. Furthermore, the medium can flow more freely around the organoid in the proposed system, potentially enhancing long-term viability and culture stability. The flexibility in electrode positioning also broadens the applicability of the system to multiple fused organoids (i.e., assembloids) (Miura et al., 2022). In the current prototype, each probe contained eight vertically aligned electrodes, with a vertical spacing of approximately 1.5 mm/8 = 0.19 mm. In the future, configurations such as four electrodes per probe across 16 probes, could yield more uniform spacing—for instance, 0.59 mm circumferentially and 0.38 mm vertically. However, this configuration would double the number of positioning manipulators, necessitating additional mechanical complexities and costs. The current 8 × 8 configuration represents a trade-off between achieving high-density spatial coverage and maintaining mechanical feasibility. Future iterations could explore designs with multiple probes per manipulator or more compact manipulators to overcome these challenges.

The system has several limitations. One significant challenge is the skilled manual labor required for assembly and, particularly, for the *in-situ* positioning of the probes and organoid. This manual alignment is prone to error due to factors such as the free movement of the organoid in the medium, the compliance of the flexible FPC electrodes, and the precision of hand adjustments. These factors currently limit the positional reproducibility of the electrodes. Simplifying these processes is essential to promote broader adoption and reduce production costs. Furthermore, medium exchange requires removing the system from the incubator and performing manual replacement. Integrating an automated alignment system using image recognition and motorized actuators, coupled with a microfluidic perfusion system for automated medium exchange, would greatly enhance the system’s usability, reproducibility, and suitability for stable, long-term experiments with minimal manual intervention. In long-term measurements, changes in activity location (i.e., channels with spikes) may reflect shifts in the biological state of the organoid. Potential contributing factors include variations at the electrode–organoid interface or the internal reorganization of neuronal connectivity. Electrical stimulation studies can help clarify connectivity patterns by identifying functional pathways within organoids.

Overall, the demonstrated system successfully recorded organoid activity with high stability over extended periods, providing a robust platform for investigating long-term neuronal development and function. This approach has the potential to advance basic neuroscience research and support the exploration of biohybrid computing and related technologies. In the short term, this platform can facilitate the structural and functional analyses of 3D neuronal networks, pharmacological testing, and studies of basic synaptic plasticity by providing access to a wide range of recording sites. In the long term, these capabilities could support the development of biohybrid computing architectures that leverage the low-power efficiency characteristics of biological neural networks.

## Data Availability

The raw data supporting the conclusions of this article will be made available by the authors, without undue reservation.
