# Supplementary material for: 360° size-adjustable microelectrode array system for electrophysiological monitoring of cerebral organoids
Source: Front Bioeng Biotechnol. 2025 Jul 22;13:1596009. doi: 10.3389/fbioe.2025.1596009 (PMC12321758; doi:10.3389/fbioe.2025.1596009)
Supplement: Supplementary file 1 [file DataSheet1.pdf]

Supplementary information

Supplementary table 1: comparison of 3D MEAs (✓ = meets requirement, ✗ = does not)

Study	MEA Type	Surface Coverage	Size Adaptability	Sample Set-up	Long-Term Demonstration	Electrode Count	Brain Organoid Test
Musick et al., 2009	Rigid stacked 3D	✗ Three layers	✗ Fixed layout	✗ Not suitable for organoid	✓ **Weeks**	✓ **58 ch**	✗ Rat cortical 3D culture
Tsai et al., 2017	CMOS-2D	✗ Single-sided	✗ Fixed layout	✓ **Simple placement**	✓ **Months**	✓ **59,760 ch**	✗ 2D slice / culture
Kalmykov et al., 2019	Self-rolling cylinder	✓ **360°**	Slightly conformable	✗ Manual insertion	✗ Short-term	✗ 12 ch	✗ Cardiac spheroid
Park et al., 2021	Buckling 3D frame	✓ **360°**	Slightly conformable	✓ **Simple drop-in to shell electrode**	✓ **Weeks**	✗ 25 ch	✓
Huang et al., 2022	Self-folding shell	✗ 4-point surround	Slightly conformable	✗ Manual insertion into shell	✓ **Weeks**	✗ 3 ch	✓
McDonald et al., 2023	Mesh MEA	✗ Single internal plane	✗ Fixed layout	✓ **Simple placement**	✓ **Weeks**	✓ **61 ch**	✓
Andrews et al., 2024	CMOS-2D	✗ Single-sided	✗ Fixed layout	✓ **Simple placement**	✗ Short-term	✓ **26k ch**	✗ Human hippocampal slice
Kim et al., 2025	Stretchable MEA	✗ Bottom-side	Slightly conformable	✓ **Drop-in microwell**	✓ **Days–weeks**	✗ 19 ch	✓
Li et al., 2025	CMOS-2D	✗ Single-sided	✗ Fixed layout	✓ **Simple placement**	✗ Short-term	✓ **236,880 ch**	✓
**This work**	360° adjustable probe array	✓ **360°**	✓ **1–3.7 mm**	✗ Manual probe alignment	✓ **Weeks**	✓ **64 ch**	✓

* A "✓" indicates that the study meets the following:
- Surface coverage: 360° coverage of the sample
- Size adaptability: Electrode layout adjustable for multi-fold size changes
- Sample set-up: Sample can be placed easily by a non-expert
- Long-term demonstration: Demonstrated for several weeks or more
- Electrode count: At least 30 recording channels
- Brain organoid test: Validated with human brain organoids/spheroids

References

Huang, Q., Tang, B., *et al.* (2022). Shell microelectrode arrays (MEAs) for brain organoids. *Sci. Adv.* 8, eabq5031. doi: 10.1126/sciadv.abq5031.

Kalmykov, A., Huang, C., Bliley, J., Shiwarski, D., Tashman, J., Abdullah, A., *et al.* (2019). Organ-on-e-chip: Three-dimensional self-rolled biosensor array for electrical interrogations of human electrogenic spheroids. *Sci. Adv.* 5, eaax0729. doi: 10.1126/sciadv.aax0729.

Kim, K., Lee, Y., Jung, K. B., Kim, Y., Jang, E., Lee, M.-O., *et al.* (2025). Highly stretchable 3D microelectrode array for non-invasive functional evaluation of cardiac spheroids and midbrain organoids. *Adv. Mater.* 37, 2412953. doi: 10.1002/adma.202570046.

Li, Q., Zhang, X., Wang, J., Müller, J., Hierlemann, A., Wu, Y., *et al.* (2025). Advanced neural activity mapping in brain organoids via field potential imaging with ultra-high-density CMOS microelectrode arrays. *bioRxiv* preprint. doi: 10.1101/2025.05.24.655914.

McDonald, M., Sebinger, D., Brauns, L., Gonzalez-Cano, L., Menuchin-Lasowski, Y., Mierzejewski, M., *et al.* (2023). A mesh microelectrode array for non-invasive electrophysiology within neural organoids. *Biosens. Bioelectron.* 228, 115223. doi: 10.1016/j.bios.2023.115223.

Musick, K., Khatami, D., Wheeler, B. C. (2009). Three-dimensional micro-electrode array for recording dissociated neuronal cultures. *Lab Chip* 9, 2036–2042. doi: 10.1039/b820596e.

Park, Y., Franz, C. K., Ryu, H., Luan, H., Cotton, K. Y., Kim, J. U., *et al.* (2021). Three-dimensional, multifunctional neural interfaces for cortical spheroids and engineered assembloids. *Sci. Adv.* 7, eabf9153. doi: 10.1126/sciadv.abf9153.

Stoppini, L., Heuschkel, M. O., Loussert-Fonta, C., Gomez Baisac, L., Roux, A. (2024). Versatile micro-electrode array to monitor human iPSC-derived 3D neural tissues at the air–liquid interface. *Front. Cell. Neurosci.* 18, 1389580. doi: 10.3389/fncel.2024.1389580.

Tsai, D., Sawyer, D., Bradd, A., Yuste, R., Shepard, K. L. (2017). A very large-scale microelectrode array for cellular-resolution electrophysiology. *Nat. Commun.* 8, 1802. doi: 10.1038/s41467-017-02009-x.
